# Low-dose interleukin-2 therapy in systemic lupus erythematosus

**DOI:** 10.2478/rir-2023-0021

**Published:** 2023-09-27

**Authors:** Antonio La Cava

**Affiliations:** Department of Medicine, University of California Los Angeles, Los Angeles, CA 90095, USA

**Keywords:** systemic lupus erythematosus, interleukin-2, low-dose IL-2, T regulatory cells

## Abstract

In systemic lupus erythematosus (SLE), T regulatory cells (T_regs_) contribute to the inhibition of autoimmune responses by suppressing self-reactive immune cells. Interleukin (IL)-2 plays an essential role in the generation, function and homeostasis of the T_regs_ and is reduced in SLE. Several clinical studies, including randomized trials, have shown that low-dose IL-2 therapy in SLE patients is safe and effective and can reduce disease manifestations. This review discusses the rationale for the use of low-dose IL-2 therapy in SLE, the clinical responses in patients, and the effects of this therapy on different types of T cells. Considerations are made on the current and future directions of use of low-dose IL-2 regimens in SLE.

## Introduction

Over the last decade, treatment of patients with systemic lupus erythematosus (SLE) with low-dose interleukin-2 (IL-2) has emerged as a promising new therapeutic modality for the amelioration of disease manifestations without significant side effects. This approach contributes to the correction of the acquired deficit of IL-2 in SLE and associates with an improvement of the impaired activity of the CD4^+^ T regulatory cells (T_regs_), whose growth and survival depend on IL-2. This review describes the rationale behind the use of low-dose IL-2 therapy in SLE and the significant progress achieved in the field, with a historical perspective.

## IL-2

IL-2 was discovered as a T cell growth factor almost half a century ago ^[1,2]^ and has since been studied extensively for its ability to control the differentiation and homeostasis of both T effector cells and Tregs.^[3]^

IL-2 is manly produced by activated CD4^+^ T cells but can also be produced by activated CD8^+^ T cells, natural killer (NK) cells, NK T cells and dendritic cells.^[4]^ It signals through the IL-2 receptor (IL-2R), a complex that consists of three distinct chains named α (CD25), ß (CD122) and γ (CD132).^[5]^ The three chains can assemble in alternative combinations to generate low, intermediate, and high affinity IL-2 receptors on different T cell subsets. The high-affinity IL-2R is a trimer made of α (CD25), β (CD122), and γ (CD132) chains, with the α chain providing affinity for IL-2 and the β and γ chains mediating signal transduction. The chains composition- which can be expressed in different forms in different immune cell types-distinctively influences cell fate by regulating different transcriptional and metabolic programs.^[6]^ The fact that FoxP3^+^ T_regs_ constitutively express the trimeric IL-2R complex makes them more sensitive for IL-2 that natural killer (NK) cells or CD8^+^ T cells that express instead the IL-2R β/γ dimer.^[7]^

The critical role of IL-2 in immune homeostasis was already evident from early studies that demonstrated that the deletion of the IL-2 gene in mice resulted in the development of autoimmune disease, in particular ulcerative colitis.^[8]^ Autoimmune manifestations were also observed in mice deficient of the genes that encode for the IL-2 receptor α-chain (CD25) and β-chain (CD122).^[9]^

## T _REGS_

Immune cells that critically depends on IL-2 are the CD4^+^ T _regs_, which are pivotal players in the maintenance of peripheral self-tolerance though the suppression of auto-reactive immune cells and proinflammatory responses.^[10]^ T_regs_ coexpress the α-chain of the IL-2R CD25 and the transcription factor forkhead box protein 3 (FOXP3) - the master regulator of T_regs_ differentiation.^[11]^ Considering that FoxP3 in human CD4^+^ T cells can be also expressed by activated T cells without a suppressive capacity, human bona fide T_regs_ must concomitantly express high levels of CD25, FoxP3, and low/no expression of the IL-7 receptor CD127 (CD4^+^FoxP3^+^CD25^high^CD127^-^ T cells).^[12]^ Yet the expression of FoxP3 is generally considered as a key element in the identification of T_regs_, including in most of the studies that have assessed the effects of low-dose IL-2 on T_regs_.

As anticipated before, IL-2 is indispensable for the development, function and survival of the T_regs_.^[13]^ Since T_regs_ are unable to produce IL-2, they fully depend on the available IL-2 produced by other cells. As a consequence, a reduced availability of IL-2- or an abnormal CD25 signaling – lead to an impairment of the T_regs_, with subsequent inefficient control of inflammation and autoimmunity.^[14]^

It has to be noted that T_regs_ are exquisitely receptive to IL-2 stimulation because of their elevated cell surface expression of the high affinity IL-2 receptor (IL-2R) CD25. The capability of the T_regs_ to respond to relatively low levels of IL-2 (that are insufficient to activate other immune cells) endows the T_regs_ with the significant advantage of maximizing usage of the IL-2 available in the microenvironment.^[15]^ After the IL-2R has been engaged, cell signaling involves the phosphorylation of the Janus-Activated Kinase (JAK) 1 and JAK3 that activate the downstream mitogen-activated protein kinase (MAPK) and phosphoinositide 3-kinase (PI3K) pathways, leading to activation and nuclear translocation of STAT5 ^[16] (^JAK inhibitors are currently being tested in clinical trials in SLE).^[17]^ The binding of signal transducer and activator of transcription 5 (STAT5) to the Foxp3 locus in response to IL-2 induces the expression of the Treg master regulator FoxP3,^[18]^ which is important not only during the initial stages of activation but also for a long-term maintenance of functional T_regs_.^[13]^

Functionally, T_regs_ adopt multiple mechanisms for the suppression of effector immune responses. In SLE, T_regs_ can inhibit the activity of T cells that help B cells in the production of autoantibodies,^[19]^ and can directly suppress autoantibody-producing B cells through the release of granzyme B and perforin ^[20]^ or via induction of anergy.^[21]^

T_regs_ also indirectly regulate the function of antigen-presenting cells (APCs) by altering antigen presentation and costimulation ^[22,23]^ through the removal of cell surface molecules on the APCs by trogocytosis.^[24]^

## Types of T _REGS_

T_regs_ that arise from the thymus are called natural T_regs_ or thymic T_regs_ (tT_regs_); T_regs_ that differentiate in the periphery are named peripheral T_regs_ (pT_regs_). T_regs_ can also be induced in vitro (induced T_regs_, iT_regs_) from CD4^+^CD25^-^ T cells following stimulation with IL-2 and transforming growth factor-β (TGF-β).^[25]^ Those iT_regs_ are unstable and can convert back into non-suppressive T effector cells, particularly in the presence of a proinflammatory microenvironment.^[26]^ Therefore, although easy to expand in vitro in numbers large enough for the transfusion into SLE patients,^[27]^ iT_regs_ are not considered an optimal choice for long-term maintenance of immune cell homeostasis driven by stable T_regs_.

Other types of peripheral immunoregulatory T cells include Tr1 and Th3 cells that, however, have different phenotypic and functional characteristics from classical T_regs_.^[28]^

A specialized population of T_regs_ has the ability to counteract the T follicular helper (Tfh) cells that communicate and activate follicular B cells in the germinal centers (GCs) for the production of high-affinity (auto) antibodies. Those T_regs_ upregulate CXCR5 and B-cell lymphoma 6 (BCL6) to migrate into the B cell follicles and are called T follicular regulatory (Tfr) cells.^[29]^ Interestingly, the homeostatic balance between Tfh cells and Tfr cells is disrupted in SLE, with a low Tfr/Tfh ratio.^[30]^ This contributes to an expansion of Tfh cells that in SLE correlates with disease severity and high titers of autoantibodies.^[31]^ Of note, IL-2 suppresses the master regulator of Tfh cell differentiation BCL6^[32]^ and therefore IL-2 inhibits the differentiation of Tfh cells,^[33]^ including in SLE.^[34]^ However, while under normal conditions the usage of IL-2 by T_regs_ (and dendritic cells) reduces IL-2 availability to Tfh cells around the B cell follicles,^[34]^ this is not the case in SLE because of the deficit of IL-2 in the disease.^[35]^ This reduction of available IL-2 in SLE is also exacerbated by the presence of circulating soluble IL-2R that competes with membrane-bound CD25 in sequestering IL-2.^[36]^

## T _REGS_ and SLE

In lupus-prone mice, the numbers of T_regs_ gradually decline as the disease progresses.^[37]^ This spontaneous decay can be prevented with the administration of low-dose IL-2, and the IL-2-driven expansion of T_regs_ associates with improved disease manifestations.^[38, 39]^ The protection from disease that associates with the expansion of the pre-existing pool of T_regs_ appears to be due to the fact that lupus T_regs_ do not have altered immunosuppressive function.^[40]^ Therefore, the insufficient capacity of the lupus T_regs_ to effectively suppress self-reactive immune cells because of the acquired deficiency of IL-2 can be amended.^[41,42]^

## Low-Dose Il-2 Therapy in SLE

Taken together, the observations reported above indicate the existence of a causal link between IL-2 shortage, Treg disruption, and immune dysregulation in SLE, implying that IL-2 supplementation has the potential to help correct these abnormalities. These findings laid the rationale for the use of low-dose IL-2 in SLE, which had been shown to be therapeutically effective in graft-versus-host disease (GHVD), type 1 diabetes, and vasculitis.^[43]^ A chronological summary of the clinical trials using low-dose IL-2 is reported in Table 1.

**Table 1:** A chronological summary of the clinical trials using low-dose IL-2

Type of trial	Objective (s)	Treatment (subjects/group)	Route administration and dose of IL-2	Outcome (s)	S.A.E.	Ref.
Single-center, open-label study in patients with lupus nephritis	Safety, efficacy, clinical outcomes	Low-dose IL-2 + S.O.C (*n* = 18) *vs*. S.O.C. only (*n* = 12)	3 cycles of 1 million IU s.c. every other day for 2 weeks followed by a 2-week break	Higher remission rate and improved renal outcomes *vs*. baseline	none	[51]
Multicenter prospective, open-label phase I/IIa clinical basket trial	Safety and efficacy, immunomonitoring	Low-dose IL-2 + S.O.C. (*n* = 6)	1 million IU/day s.c. for 5 days followed by fortnightly injections of 1 million IU/day for 6 months	Treg expansion, improved Clinical Global Impression score	none	[53]
Single-center, uncontrolled, phase I/IIa clinical trial in patients with refractory SLE	Safety, efficacy, clinical out- comes	Low-dose IL-2 + S.O.C. (*n* = 12)	4 cycles of 0.75, 1.5, or 3.0 million IU/day s.c. for 5 days followed by a 9–16 day rest. Dose adaption according to predefined criteria	Reduction in SLEDAI score at day 62. No severe disease flares and improvement/resolution of disease manifestations correlating with an increase in Tregs	none	[47]
Single-center, uncontrolled study in patients with refractory SLE	Efficacy, clinical outcomes	Low-dose IL-2 + rapamycin (*n* = 50)	100 WIU s.c. at 3–5 days per month combined with rapamycin (0.5 mg, once every other day, orally) for 24 weeks	Reduction in SLEDAI score after 6, 12 and 24 weeks; reduction in prednisone dose, increase in Treg numbers	none	[54]
Single-center, randomized, double-blind, placebo-controlled phase II clinical trial	Safety, efficacy	Low-dose IL-2 + S.O.C. (*n* = 30) *vs*. placebo + S.O.C. (*n* = 30)	3 cycles of 1 million IU s.c. every other day for 2 weeks followed by a 2-week break as one treatment cycle	Better SRI-4 response rates *vs*. placebo at weeks 12 and 24. Higher complete remission in patients with lupus nephritis *vs*. placebo	none	[49]
Multicenter, double-blind, randomized, placebo-controlled phase II clinical trial in active SLE	Safety, efficacy biological responses	Low-dose IL-2 + S.O.C. (*n* = 50) *vs.* placebo + S.O.C. (*n* = 50)	1.5 million IU s.c. for 5 days followed by weekly 1.5 million IU s.c. from day 8 to week 12	Only post hoc analysis that excluded patients from two sites showed a better SRI-4 response rate *vs*. placebo	3 among IL-2; 2 among placebo	[52]

S.A.E., serious adverse event; SLEDAI, Systemic Lupus Erythematosus Disease Activity Index; S.O.C, standard-of-care treatment; SRI-4, SLE Responder Index-4.

The first reported successful use of low-dose recombinant human IL-2 (rIL-2) was in a patient with active SLE who received four treatment cycles of subcutaneous low-dose rIL-2 (1.5 million IU [international unit] each cycle except the second cycle that was 3 million IU) separated by 9–16 day resting periods.^[44]^ The rIL-2 was aldesleukin – a biologic response modifier approved in several European countries and in the USA for the treatment of cancers.^[45]^ The treatment associated with an expansion of functional T _regs_, a significant reduction in the levels of circulating anti-dsDNA antibodies, and the subsidence of arthritis and skin eruptions.^[44]^

Expansion of peripheral T_regs_ following low-dose IL-2 therapy was confirmed in a subsequent study in which five patients with refractory SLE received daily subcutaneous injections of 1.5 million IU aldesleukin for five consecutive days. However, the study only evaluated T_regs_ and not clinical parameters of SLE disease activity.^[46]^

The first proof-of-concept, single-center phase 1/2a trial started in 2014 and was done on a total of 12 patients with active and refractory SLE to evaluate safety, tolerability, and clinical responses to treatment with four 5-day cycles of low-dose rIL-2 (aldesleukin) in addition to standard medical care.^[47]^ Patients were started with a daily dose of 1.5 million IU rIL-2 in the first treatment cycle and then the subsequent daily doses were either increased to 3 million IU or reduced to half of the previous dose. All patients had a dose-dependent increase in T_regs_ after each treatment cycle and 11 patients achieved the primary endpoints of at least a 100% increase in the proportion of T_regs_ after four treatment cycles. This correlated with decreased safety of estrogens in lupus erythematosus national assessment–SLE disease activity index (SELENA-SLEDAI) scores (secondary endpoint). Clinical responses with a meaningful improvement of clinical manifestations such as arthritis, rash, or alopecia were also observed in two third of the patients after the four treatment cycles. The trial showed that a safe dose of aldesleukin was 0.75–1.5 million IU/day, and that adverse events manifested with a daily dose was 3 million IU^[47]^

Safety of use of low-dose IL-2 in SLE patients was also reported in two larger clinical trials.^[48, 49]^ The first trial was a prospective, open-label study on thirty-eight SLE patients with active SLE who received three cycles of 1 million IU recombinant human IL-2 (rIL-2) subcutaneously every other day for 2 weeks followed by a two-week washout period. Almost 90% of the patients achieved SLE Response Index (SRI)-4 response at the end of 12 weeks, accompanied by significant reductions in SELENA-SLEDAI and a reduction of glucocorticoid dose > 50% as compared with baseline in over two-third of the patients.^[48]^

The other study was the first randomized, double-blind, placebo-controlled, single-center phase 2 trial.^[49]^ Sixty patients with active SLE were randomly assigned in a 1: 1 ratio to receive either three cycles of low-dose IL-2 therapy or placebo on top of standard-of-care therapy. The primary endpoint was the proportion of patients who achieved a SLE responder index-4 (SRI-4) response at week 12 compared with placebo. The IL-2 group had early significantly higher proportions of SRI-4 responders than the placebo group and up to week 24 (29% difference) and 54% of the patients in a subgroup with lupus nephritis had a complete renal response at week 12 *vs.* 8% in the placebo group. Moreover, the IL-2 group had reduced proteinuria, increased C3 and C4 complement factors and decreased anti-dsDNA-antibodies that associated with a transient but significant expansion of the peripheral T _regs_. The treatment was well tolerated, without major adverse events.^[49]^

Positive effects in patients with refractory lupus nephritis were also seen in another study that reported a dramatic reduction of proteinuria, of urine erythrocytes, and anti-dsDNA antibodies after starting low-dose IL-2 therapy, with no flares during the second year of IL-2 maintenance therapy.^[50]^

Another single-center, open-label clinical study used three cycles of low-dose IL-2 in addition to standard-of-care therapy in eighteen patients with active lupus nephritis. Twelve patients who only received standard-of-care therapy served as controls. The results showed that the IL-2 group had remission rates > 3-fold higher than the control group after 10 weeks of treatment, in addition to more pronounced decrease in proteinuria and haematuria that accompanied with an expansion of T_regs_.^[51]^

More recently, results have been reported from an international, double-blind, placebo-controlled phase 2 clinical trial (LUPIL-2) with low-dose IL-2 therapy (aldesleukin, ILT-101) in moderately-to-severely active SLE patients.^[52]^ This multi-center trial included 36 sites from 10 different countries and aimed to assess safety, clinical efficacy, and biological responses. Hundred patients were randomly assigned in a 1: 1 ratio to receive subcutaneously either 1.5 million IU IL-2/day or placebo for five consecutive days followed by a maintenance period of weekly injections of 1.5 million IU IL-2 or placebo from day 8 to week 12. The primary endpoint was the SRI-4 response at week 12. Secondary endpoints were the differences in SELENA-SLEDAI scores from baseline to week 12, SRI-4 responses at week 8, SRI-6 and SRI-8 responses at week 12, time-to-first SRI-4 response, changes in glucocorticoid doses between baseline and week 12, and the proportions of patients in clinical remission at week 12 (*i.e.*, with a SELENA-SLEDAI score < 2). Patients fulfilling the SRI-4 response criteria at week 12 were eligible to receive IL-2 or placebo for another 12 weeks in a blinded fashion until week 24. The results showed an expansion of functional T_regs_ in the IL-2 group and not in the placebo group. The therapy was well tolerated and only associated with transient and mild to moderate adverse events, being mild injection site reactions the most frequent side effect. Of note, the treatment did not lead to the generation of anti-IL-2 antibodies. However, the primary endpoint was not met since the IL-2 group had an SRI-4 response rate of 68% *vs*. 58% in the placebo group in the intention-to-treat (ITT) population. However, post hoc analyses identified an unexplained SRI-4 response rate of 100% in 28% of patients receiving placebo in two sites of one country. Therefore, it was decided to exclude patients receiving IL-2 (27% of the total number of patients) or placebo from those two sites. Such exclusion led to the primary endpoint being met, with statistically significant differences between the SRI-4 response rate in the IL-2 (83.3%) *vs*. the placebo group (51.7%). Significant differences were also observed for the secondary endpoints of decreased SELENA-SLEDAI scores between baseline and week 12 for the IL-2 group, reduced daily glucocorticoid dose at week 12, higher proportions of SRI-6 and SRI-8 responders, remissions, and a shorter time-to-first SRI-4 response in the IL-2 group. Amelioration of rash, mucosal ulcers, alopecia and arthritis were also observed preferentially in the IL-2 group at week 12.^[52]^

## Mechanisms of Action

There is consistency among reports that low-dose IL-2 therapy is well tolerated and associates with the expansion of T_regs_ (also in conditions different from SLE, as shown in a multi-center clinical basket trial with forty-six patients with multiple diseases that included six SLE patients).^[53]^ However, the expansion of T_regs_ appears not to be the only factor affected by therapy with low-dose IL-2. For example, a prospective open-label study of fifty patients with refractory SLE who received low-dose IL-2 for 3 to 5 days monthly together with 0.5 mg of the immunosuppressive drug rapamycin on alternate days, the reduced prednisone dosage and reduced SLEDAI scores up to 24 weeks post-treatment not only associated with an expansion of T_regs_ but also with a decreased Th17/T_regs_ ratio.^[54]^ Other studies had shown that not only had low-dose IL-2 led to quantitative and functional improvement of T_regs_, but also associated with a reduction of both Th17 cells and Tfh cells.^[48,49]^ This inhibition of Tfh cells by IL-2 has been demonstrated in lupus mice,^[34]^ and the therapeutic efficacy of low-dose IL-2 has been linked to the inhibition of Tfh cells.^[55]^ Thus, the reduced disease activity in SLE patients treated with low-dose IL-2 associates not only with a boosting of the suppressive T_regs_ but also with the inhibition of proinflammatory Th17 and Tfh cells ^[48,49]^ and an increased ratio of Tfr: Tfh cells.^[56]^ However, it has still to be defined the exact contribution of IL-2 to each of these findings in relation to the overall outcomes. If both T_regs_ and/or Tfr cells (for facilitation of immune regulation) and Tfh cells (for suppression of proinflammatory responses) need to be targeted simultaneously, one should take into account relative contributions of each subset, since the preferential usage of IL-2 by T_regs_ is known to limit IL-2 availability to Tfh cells. This consideration has been the basis of recent developments in targeting IL-2 specifically to T_regs_ or Tfh cells with engineered IL-2 mimetic variants (muteins) that can selectively bind the intermediate IL-2R or the high-affinity IL-2R.^[57,58]^

In this regard, molecules with higher affinity for the trimeric IL-2R than for dimeric IL-2R can better target T_regs_ and have a longer half-life as compared to IL-2, so they have longer lasting effects. One of these muteins, named NKTR-358 (whose conjugation with polyethylene glycol extends its half-life to 7–13 days), has been studied in a randomized, placebo-controlled phase 1 study that included thirty-six SLE patients with mild-to-moderate disease activity and seventy-six healthy volunteers.^[59]^ The treatment with single or multiple ascending doses was well tolerated and resulted in a dose-dependent expansion of T_regs_ and improved cutaneous lupus disease manifestations in about 39% patients with cutaneous involvement. A confirmatory randomized, placebo-controlled phase 2 trial is ongoing.

## Conclusions

Differently from the high-dose IL-2 protocols used for the treatment of cancer patients (*i.e.*, > 10^8^ IU/day) that associate with elevated toxicity,^[60]^ the use of low-dose IL-2 in SLE is well-tolerated and beneficial on multiple disease manifestations and does not associate with generalized immune suppression.^[61]^ Therefore, low-dose IL-2 could become a valuable synergizing complement to current therapies in SLE (although likely insufficient as monotherapy to halt a complex disease such as SLE). Yet the short half-life of IL-2 poses questions on whether repeated injections in short durations may be required, to maintain efficacy. Also, longterm data will clarify whether prolonged treatments with low-dose IL-2 may cause toxicity and help define what is the optimal number of T_regs_ required to rebalance immune homeostasis while avoiding immune suppression, in addition to defining how to properly tune them, together with Tfh and possibly Tfr cells.
